# l-Cysteine and l-Serine Modified Dendrimer with Multiple Reduced Thiols as a Kidney-Targeting Reactive Oxygen Species Scavenger to Prevent Renal Ischemia/Reperfusion Injury

**DOI:** 10.3390/pharmaceutics10040251

**Published:** 2018-12-01

**Authors:** Satoru Matsuura, Hidemasa Katsumi, Hiroe Suzuki, Natsuko Hirai, Rie Takashima, Masaki Morishita, Toshiyasu Sakane, Akira Yamamoto

**Affiliations:** 1 Department of Biopharmaceutics, Kyoto Pharmaceutical University, Yamashina-ku, Kyoto 607-8414, Japan; kd15010@poppy.kyoto-phu.ac.jp (S.M.); animal_0_estrellas2@yahoo.co.jp (H.S.); ky13275@poppy.kyoto-phu.ac.jp (N.H.); ky14206@poppy.kyoto-phu.ac.jp (R.T.); morishita@mb.kyoto-phu.ac.jp (M.M.); sakane@kobepharma-u.ac.jp (T.S.); yamamoto@mb.kyoto-phu.ac.jp (A.Y.); 2 Department of Pharmaceutical Technology, Kobe Pharmaceutical University, Higashinada-ku, Kobe 658-8558, Japan

**Keywords:** dendrimer, drug delivery, renal targeting, kidney, amino acid, reactive oxygen species scavenger, ischemia/reperfusion injury

## Abstract

l-cysteine (Cys)- and l-serine (Ser)-modified, third-generation polyamidoamine (PAMAM) dendrimer with multiple reduced thiols (Ser-PAMAM-Cys) was synthesized as a kidney-targeting reactive oxygen species (ROS) scavenger to help prevent renal ischemia/reperfusion injury. Ser-PAMAM-Cys effectively scavenged 2,2-diphenyl-1-picrylhydrazyl (DPPH) radical and ROS (hydrogen peroxide and hydroxyl radical) in phosphate-buffered saline (PBS). In addition, ~64% of ^111^In-labeled Ser-PAMAM-Cys accumulated in mouse kidney 3 h after intravenous administration. An in vivo imaging system (IVIS) study indicated that near-infrared fluorescence dye (NIR)-labeled Ser-PAMAM-Cys specifically accumulated in the kidney. In a mouse renal ischemia/reperfusion injury model, increases in the kidney damage markers creatinine (Cre) and blood urea nitrogen (BUN) were significantly inhibited by intravenous Ser-PAMAM-Cys administration. In contrast, Cys injection had no statistically significant effect of preventing Cre or BUN elevation relative to the control. Ser-PAMAM-Cys also effectively downregulated the inflammatory factors NGAL, IL-18, ICAM-1, and VCAM-1 in the renal ischemia/reperfusion injury model. These results indicate that Ser-PAMAM-Cys is a promising kidney-targeting ROS scavenger which could prevent ischemia/reperfusion-induced renal failure.

## 1. Introduction

Renal ischemia/reperfusion injury may result in acute kidney failure, delayed renal function, and early mortality after kidney transplantation and hemorrhagic shock [1,2,3]. During reperfusion after renal ischemia, reactive oxygen species (ROS) are readily generated in the kidney and cause oxidative damage [4]. ROS induce intercellular adhesion molecule-1 (ICAM-1) and vascular cell adhesion molecule-1 (VCAM-1) which recruit leukocytes to the region with ischemia/reperfusion injury and cause a secondary disorder [5]. Therefore, ROS are associated with renal ischemia/reperfusion injury. Antioxidants which scavenge ROS may serve as therapeutic agents preventing renal ischemia/reperfusion injury. Reduced thiols like *N*-acetyl cysteine and glutathione efficiently scavenge ROS [6,7,8,9]. Sehirili et al. reported that low molecular weight reduced thiols effectively suppressed increases in the levels of creatinine (Cre) and blood urea nitrogen (BUN) after ischemia/reperfusion [6]. However, they are also rapidly eliminated from circulation and either metabolized in the liver or excreted in the urine. Moreover, large quantities of reduced thiols were required to prevent renal ischemia/reperfusion injury [10]. To maximize their effect, it is necessary to control their pharmacokinetics using drug delivery technologies. Conjugating reduced thiols to macromolecular drug carriers may effectively control their delivery [11,12,13]. We recently developed the l-serine (Ser)-modified polyamidoamine (PAMAM) dendrimer. It is a highly potent renal-targeting drug carrier [14]. In mice, ~82% of the intravenously administered Ser-PAMAM dose accumulated in the kidney. Therefore, the conjugation of reduced thiols to Ser-PAMAM may target the delivery of reduced thiols to the kidney and prevent renal ischemia/reperfusion injury.

The aim of the present study was to develop a kidney-targeting reduced thiol conjugated to Ser-PAMAM to prevent renal ischemia/reperfusion injury. To this end, we conjugated Cys and Ser to PAMAM (Ser-PAMAM-Cys). The Cys and Ser were covalently bound to the PAMAM amine moiety as a reduced thiol and a renal targeting ligand, respectively. Next, the ROS- and free radical-scavenging ability and renal targeting efficacy of Ser-PAMAM-Cys were investigated. Finally, we examined the efficacy of Ser-PAMAM-Cys at preventing ROS-mediated kidney failure in a mouse renal ischemia/reperfusion injury model.

## 2. Materials and Methods

### 2.1. Materials

PAMAM (PAMAM dendrimer with an ethylenediamine core (generation 3)) in methanol (20% *w*/*w*) was purchased from Sigma-Aldrich (St. Louis, MO, USA). Boc-Ser(*t*Bu)-OH and HOBt were purchased from Watanabe Chemical Industries (Hiroshima, Japan). Boc-Cys(Trt)-OH and HBTU were purchased from Merck Millipore (Billerica, MA, USA). Dimethyl sulfoxide (DMSO), *N*,*N*-dimethylformamide (DMF; superdehydrated grade), diethyl ether (Et_2_O; superdehydrated grade), *N*,*N*-diisopropylethylamine (DIPEA), piperidine, trifluoroacetic acid (TFA), and 2,2-diphenyl-1-picrylhydrazyl (DPPH) were purchased from Wako Pure Chemical Industries (Osaka, Japan). PD-10 was purchased from GE Healthcare Japan (Tokyo, Japan). The ^111^InCl_3_ was kindly donated by Nihon Medi-Physics (Tokyo, Japan). DTPA anhydride was purchased from Chemical Dojin Co. Ltd. (Kumamoto, Japan). All other chemicals were commercial reagent grade.

### 2.2. Animals

Male ddY mice (5 wks, 25 g) and male C57BL/6J mice (6 wks, 20–25 g) were purchased from Japan SLC (Shizuoka, Japan). Animals were maintained under conventional housing conditions. All animal experiments were conducted according to the principles and procedures outlined in the National Institutes of Health Guide for the Care and Use of Laboratory Animals. The Animal Experimentation Committee of the Kyoto Pharmaceutical University approved all experimental protocols involving animals (17-025, 17-028, 17-029 from April.2017).

### 2.3. Synthesis and Characterization of Serine and Cysteine Modified PAMAM Dendrimers

Ser-PAMAM-Cys was synthesized by reacting Ser and Cys with third-generation (G3) PAMAM according to the HBTU-HOBt method [14]. Briefly, PAMAM dendrimer (86.5 mg, 0.013 mmol) was coupled with 0.22 eq (equivalent to the dendrimer surface amino group) Boc-Cys(Trt)-OH (40.9 mg, 0.088 mmol) and 0.88 eq Boc-Ser(*t*Bt)-OH (92.1 mg, 0.35 mmol) in DMF/DMSO (1:1) by mixing it with 1.1 eq HBTU (167 mg, 0.44 mmol), 1.1 eq HOBt (59.6 mg, 0.44 mmol), and 2.2 eq DIPEA(154 µL, 0.88 mmol). The reaction mixtures were incubated at room temperature until a negative ninhydrin test result on thin layer chromatography (TLC) was obtained. The coupled solution was purified by precipitation 3× with Et_2_O. The precipitates were dissolved in a mixture of 95% TFA, 2.5% TIS, and 2.5% water to deprotect the Boc, tBu, and Trt groups. The reaction mixtures were then incubated at room temperature for 90 min. After deprotection, the solution was purified by precipitation 3× with Et_2_O. The crude precipitates were dissolved in ultrapure water and passed through a PD-10 column to separate the products by size exclusion chromatography. These were then lyophilized to obtain Ser-PAMAM-Cys. The reduced thiol groups derived from Cys on PAMAM were detected by Ellman’s method [15]. The number of Cys on Ser-PAMAM-Cys was estimated to be ~6.4 (Cys content: 20%). The mean diameter and ζ-potential were measured at 1 mg mL^−1^ in phosphate-buffered saline (PBS, pH 7.4) with a Zetasizer Nano (Malvern Panalytical, Malvern. UK) at 25 °C. Ser-PAMAM-Cys was identified by matrix-assisted laser desorption/ionization time-of-flight mass spectrometry (MALDI-TOF MS) (Microflex, Bruker, Germany). To confirm the purity and stability of Ser-PAMAM-Cys, products (lyophilized and stored for two years at 15–25 °C) were evaluated by 15% polyacrylamide gel electrophoresis on sodium dodecyl sulfate (SDS-PAGE) under nonreducing conditions.

### 2.4. Hydrogen Peroxide-Scavenging Ability of Ser-PAMAM-Cys

The hydrogen peroxide-scavenging ability of Ser-PAMAM-Cys was evaluated by the BES-H_2_O_2_ probe method with a slight modification [16]. In brief, Ser-PAMAM-Cys and Cys were added to 1,000 µM hydrogen peroxide at concentration of 250 µM (as the thiol concentrations in PBS) and incubated at 37 °C for 1 h in the dark. About 20 µL each of the mixture and 5 µM BES-H_2_O_2_ were reacted for 30 min. The fluorescence intensity of these samples was measured in a microplate reader (PowerScan HT, BioTek Instruments, Inc., Winooski, VT, USA) at an excitation wavelength of 485 nm and an emission wavelength of 535 nm.

### 2.5. Hydroxyl Radical-Scavenging Ability of Ser-PAMAM-Cys

The hydroxyl radical-scavenging ability of Ser-PAMAM-Cys was evaluated by the luminol probe method with a slight modification [17]. A 1 mM luminol solution was prepared in 5 mM sodium hydroxide solution. About 50 µL each of 50 µM Ser-PAMAM-Cys and Cys in PBS (as the thiol concentrations in PBS), 50 µL each of 2 mM hydrogen peroxide and 50 µL of 1 mM ferrous sulfate (both in ultrapure water) were mixed successively. The luminescence of this reaction mixture was measured for 0.1 s with a luminometer (Lumat LB9507, EG & G Berthold AG, Bad Wildbad, Germany).

### 2.6. DPPH Radical-Scavenging Ability of Ser-PAMAM-Cys

The free radical-scavenging ability of Ser-PAMAM-Cys was evaluated by the DPPH method with a modification [18]. About 100 µL of 625 µM DPPH in ethanol was added 20 µL of 50 µM Ser-PAMAM-Cys and Cys (as the thiol concentrations in PBS) then incubated at room temperature for 60 min. Absorbances were measured at 540 nm in a microplate reader (PowerScan HT, BioTek Instruments Inc., Winooski, VT, USA).

### 2.7. Tissue Distribution of Ser-PAMAM-Cys in Mice

Ser-PAMAM-Cys was radiolabeled with ^111^In by a previous method [19]. ^111^In-labeled Ser-PAMAM-Cys was intravenously administered at 1 mg kg^−1^. At predetermined times post-injection, blood and major tissues were collected under isoflurane anesthesia. Radioactivity was measured with a gamma counter (1480WizardTM3”, PerkinElmer, Inc., Waltham, MA, USA) as previously described [14]. To visualize its distribution, Ser-PAMAM-Cys was labeled with the near-infrared fluorescence (NIR) VivoTag^®^ 800 (PerkinElmer, Inc., Waltham, MA, USA) according to the manufacturer’s instructions. NIR-labeled Ser-PAMAM-Cys was intravenously administered to ddY mice. Under isoflurane anesthesia, the mice were perfused with 10 mL saline through the left ventricle to flush out dendrimers in the blood or loosely bound to tissues. The liver, kidneys, spleen, heart, and lungs were excised and rinsed with saline. Ex vivo fluorescence images were acquired with an IVIS 60 min after intravenous injection as previously described [14].

### 2.8. Prevention of Renal Ischemia Reperfusion Injury by Ser-PAMAM-Cys

A mouse renal ischemia/reperfusion injury model was established according to a previously described method [20,21]. Under isoflurane anesthesia, the left kidney was excised from the back. Renal ischemia was induced by occluding the right renal artery and vein for 30 min. Ser-PAMAM-Cys or Cys was intravenously administrated into C57BL/6J mice at 0.27 µmol thiols kg^−1^ immediately before reperfusion. After 24 h, blood was collected from the vena cava and the kidneys were excised under isoflurane anesthesia. The plasma creatinine (Cre) and blood urea nitrogen (BUN) levels were determined with a Cre measuring kit (LaboAssay^TM^, Wako Pure Chemical Industries, Osaka, Japan) and a BUN measuring kit (DIUR-100, BioAssay Systems, Hayward, CA, USA). The upper half of the excised left kidney was fixed in 4% buffered paraformaldehyde and embedded in paraffin blocks which were then sliced by microtome into 5-µm-thick sections. These were stained with hematoxylin and eosin (H&E) and renal injury was evaluated under light microscopy (Biozero, Keyence Corporation, Osaka, Japan).

### 2.9. Effect of Ser-PAMAM-Cys on Inflammatory Factors Induced by Renal Ischemia/Reperfusion

Total RNA was extracted from the lower half of the excised left kidney using RNA extraction pre-cocktails (Sepasol^®^-RNA I Super G, Nacalai Tesque, Kyoto, Japan) according to the manufacturer’s instructions. Reverse transcription was performed with a ReverTra^®^ Ace qPCR RT Master Mix with gDNA Remover (Toyobo Co. Ltd., Osaka, Japan) according to the manufacturer’s instructions. Quantitative real-time PCR (qRT-PCR) was run on a real-time PCR thermal cycler (LightCycler^®^ Nano System, Roche Diagnostics K.K., Tokyo, Japan) with SYBR Green I based premix reagent (TB GreenTM Premix Ex TaqTM II (Tli RNaseH Plus); TaKaRa Bio Inc., Kusatsu, Shiga, Japan) according to the manufacturer’s instructions. Quantification was conducted in LightCycler^®^ Nano SW v. 1.1. The primer sequences used were reported earlier [22,23,24,25,26]. The primer sequences were 5′-CACAGGTATCCTCAGAGCT-3′ and 5′-TGTAGTCCGTGGTGGCCAC-3′ for neutrophil gelatinase-associated lipocalin (NGAL), 5′-AGGCCTGACATCTTCTGCAA-3′ and 5′-TCTGACATGGCAGCCATTGT-3′ for IL-18, 5′-GGACCACGGAGCCAATTTC-3′ and 5′-CTCGGAGACATTAGAGAACAATGC-3′ for intercellular adhesion molecule-1 (ICAM-1), 5′-ACAAAACGATTGCTCAAATCGG-3′ and 5′-CGCGTTTAGTGGGCTGTCTATC-3′ for vascular cell adhesion molecule-1 (VCAM-1), and 5′-CATCCGTAAAGACCTCTATGC-3′ and 5′-ATGGAGCCACCGATCCACA-3′ for β-actin (Actb).

### 2.10. Statistical Analysis

Statistical significance was assessed by one-way ANOVA then the Tukey-Kramer Multiple Comparison Test for multiple groups at a significance level of *p* < 0.05.

## 3. Results

### 3.1. Physicochemical Properties of Ser-PAMAM-Cys

The mean diameter of Ser-PAMAM-Cys was 4.44 ± 0.23 nm. The ζ-potential of Ser-PAMAM-Cys was 13.40 ± 0.89 mV. The mass of Ser-PAMAM-Cys was 10,207 Da which corresponds to ~32 molecules of conjugated Ser and Cys (Figure S1). To evaluate the purity and stability of the products, we performed nonreducing SDS-PAGE (Figure S2). There was only one band for Ser-PAMAM-Cys (Cys content: 20% and 40%). Furthermore, we confirmed that there were no peaks in the high-molecular weight (approximately 20 kDa) corresponding to the dimer of Ser-PAMAM-Cys in the MALDI-TOF spectra (Figure S1).

### 3.2. ROS- and Radical-Scavenging Ability of Ser-PAMAM-Cys

**Figure 1:**
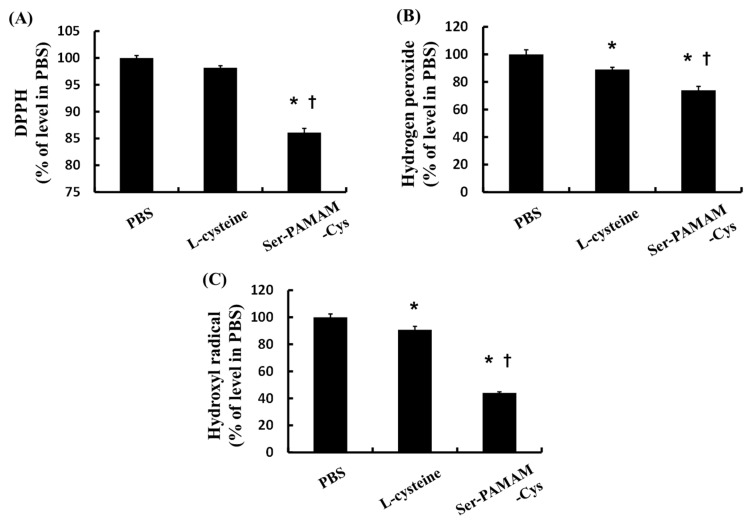
Relative abilities of l-cysteine and Ser-PAMAM-Cys to scavenge (**A**) DPPH, (**B**) hydrogen peroxide, and (**C**) hydroxyl radical. Results are expressed as means ± SE for five experiments. * *p* < 0.05: significantly different from the PBS group. ^†^ *p* < 0.05: significantly different from the l-cysteine group.

Figure 1 shows the ROS- and radical-scavenging abilities of Cys and Ser-PAMAM-Cys as the ratio of the remaining ROS and free radical relative to the PBS-treated control group. Ser-PAMAM-Cys significantly reduced DPPH, hydrogen peroxide, and hydroxyl radical relative to the control and Cys.

### 3.3. Pharmacokinetics of Ser-PAMAM-Cys

**Figure 2:**
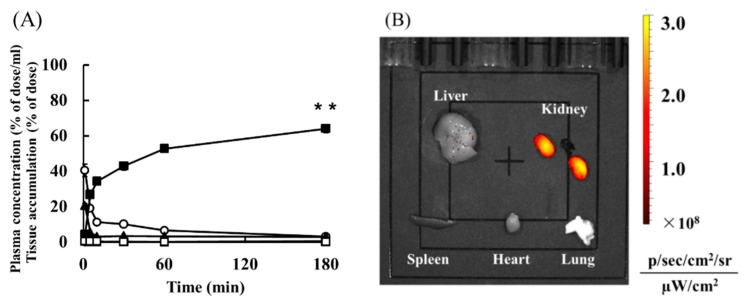
(**A**) Time courses of plasma concentration and tissue accumulation after intravenous administration of 1 mg kg^−1^ of ^111^In-labeled Ser-PAMAM-Cys. Results are expressed as means ± SE for three mice. ○, plasma; ▲, liver; ■, kidney; ◊, spleen; △, heart; □, lung. (**B**) Ex vivo imaging of NIR-labeled Ser-PAMAM-Cys 60 min after intravenous injection. Fluorescence intensities were determined for the liver, kidney, spleen, heart, and lung. ** *p* < 0.01, significantly different from other tissues.

Figure 2A shows the plasma concentration and tissue distribution of ^111^In-Ser-PAMAM-Cys after intravenous injection. The ^111^In-Ser-PAMAM-Cys rapidly disappeared from the blood. The radioactivity level in the kidney (as ^111^In-Ser-PAMAM-Cys) was 64.3% 180 min after intravenous injection.

Table 1 shows the pharmacokinetic parameters of Ser-PAMAM-Cys. The renal and hepatic uptake clearance (CL_kidney_ and CL_liver_) of Ser-PAMAM-Cys were 2.86 mL h^−1^ and 0.09 mL h^−1^, respectively. The renal uptake clearance (CL_kidney_) of Ser-PAMAM-Cys was ~81.3% of the total body clearance.

Figure 2B shows ex vivo imaging of NIR-labeled Ser-PAMAM-Cys 60 min after intravenous injection. High fluorescence intensity from NIR-labeled Ser-PAMAM-Cys was observed mainly in the kidney.

### 3.4. Ser-PAMAM-Cys Prevention of Renal Ischemia/Reperfusion Injury

**Figure 3:**
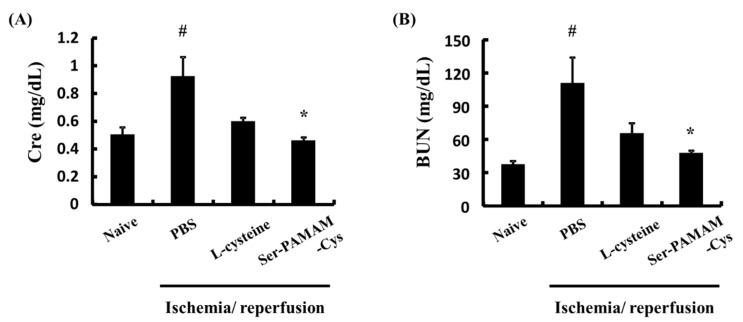
Effect of L-cysteine and Ser-PAMAM-Cys on plasma levels of (**A**) Cre and (**B**) BUN 24 h after ischemia/reperfusion. Results are expressed as means ± SE for five mice. ^#^ *p* < 0.05, significantly different from the naive group. * *p* < 0.05, significantly different from the ischemia/reperfusion + PBS group.

Figure 3 shows the effect of Ser-PAMAM-Cys on the plasma Cre and BUN concentrations 24 h after reperfusion in a mouse renal ischemia/reperfusion injury model. Plasma Cre and BUN increased to 0.92 ± 0.14 mg dL^−1^ and 110.8 ± 22.9 mg dL^−1^, respectively, after ischemia/reperfusion in the control. Therefore, renal injury was induced in this model. Cys had no statistically significant effect in preventing Cre or BUN elevation. In contrast, Ser-PAMAM-Cys administration significantly suppressed increases in the Cre and BUN levels.

**Figure 4:**
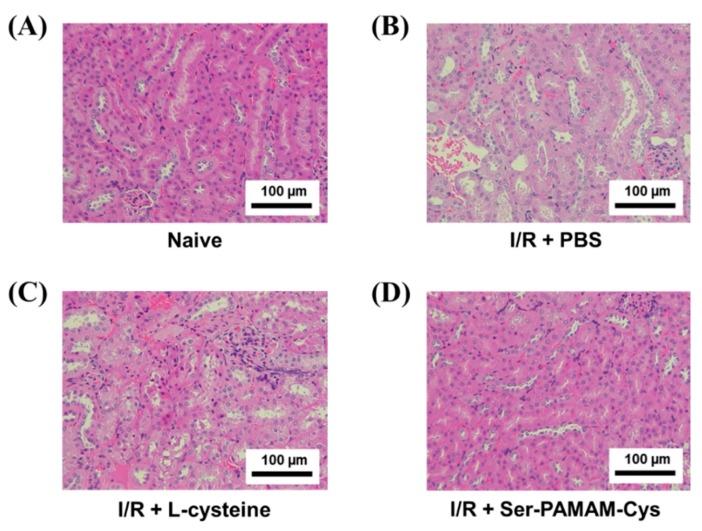
Effect of l-cysteine and Ser-PAMAM-Cys on changes in renal histology. Histological micrographs of sections from (**A**) naive and ischemia/reperfusion (I/R) mouse model kidneys after intravenous injection of (**B**) PBS, (**C**) L-cysteine, and (**D**) Ser-PAMAM-Cys. Scale bar: 100 μm.

Figure 4 shows H&E-stained renal sections from naive and treated mice. The renal tubules of the naive group were thick and well-aligned (Figure 4A). In the ischemia/reperfusion + PBS group, however, the tubules were relatively thin and there were gaps between them indicating damage (Figure 4B). Cys administration slightly mitigated the pathological changes in the renal structures compared to the control. In contrast, Ser-PAMAM-Cys administration decreased ischemia/reperfusion-induced damage and maintained the renal structure in the treated mice comparable to that of the naïve mice.

**Figure 5:**
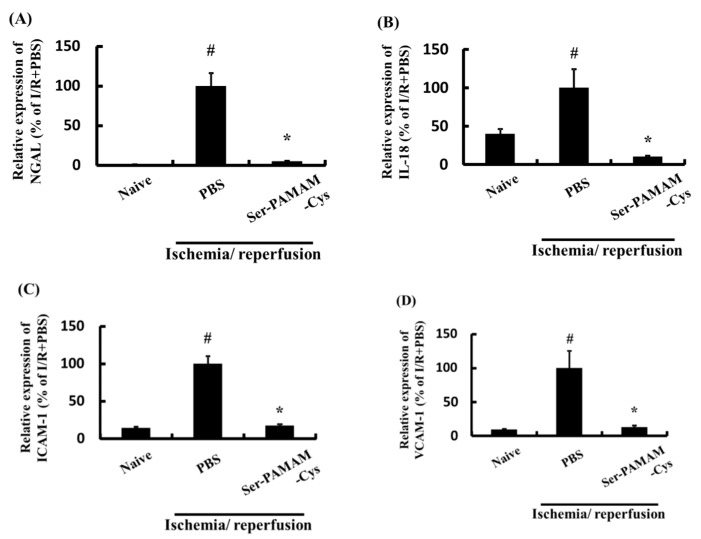
Effect of Ser-PAMAM-Cys on expression levels of (**A**) NGAL, (**B**) IL-18, (**C**) ICAM-1, and (**D**) VCAM-1 in mouse ischemia/reperfusion model kidneys. Results are expressed as means ± SE for five mice. ^#^ *p* < 0.05, significantly different from the naive group. * *p* < 0.05, significantly different from the ischemia/reperfusion (I/R) + PBS group.

Figure 5 shows the effect of Ser-PAMAM-Cys on the renal NGAL, IL-18, ICAM-1, and VCAM-1 levels after ischemia/reperfusion. They significantly increased after renal ischemia/reperfusion but were significantly downregulated by intravenous Ser-PAMAM-Cys administration.

## 4. Discussion

In the present study, we successfully synthesized Ser-PAMAM-Cys to scavenge ROS and prevent renal ischemia/reperfusion injury. We recently demonstrated that Ser-PAMAM accumulates mainly in the kidney via glomerular filtration [14]. The tissue distribution of macromolecular carriers is determined by their physicochemical properties like molecular size and surface charge [11]. In the present study, therefore, Ser-modification accounted for the efficiency of renal Ser-PAMAM-Cys distribution. The highly potent renal targeting is probably because a low degree of Cys modification (Cys content: 20%; Ser content: 80%) did not significantly influence the physicochemical properties of Ser-PAMAM or the affinity of Ser for the kidney. In contrast, Ser-PAMAM-Cys with a higher degree of Cys modification (Cys content: 40%; Ser content: 60%) had renal targeting ability but comparatively lower renal distribution (Figure S3). Taken together, these results indicate that ≥80% Ser modification is required for highly potent renal drug targeting using third-generation PAMAM.

Ser-PAMAM-Cys scavenges ROS and radicals more effectively than Cys. Low molecular weight thiols like Cys and glutathione easily self-oxidize and form intermolecular disulfide linkages. These reactions diminish their ROS- and radical-scavenging powers [27,28]. In contrast, dendrimers may prevent intermolecular disulfide linkage formation by increasing the steric distance between the thiols [12]. These results suggest that the higher thiol stability of Ser-PAMAM-Cys may contribute to its ROS- and radical-scavenging efficacy.

The preventive effect of Ser-PAMAM-Cys was proportional to its renal distribution. NGAL, IL-18, ICAM-1, and VCAM-1 are all induced by ROS in ischemia/reperfusion injury [29,30,31]. IL-18, ICAM-1, and VCAM-1 enhance leukocyte adhesion and extravasation [32,33,34]. In our previous study, we reported that Ser-PAMAM accumulates mainly in the proximal tubule which is easily damaged by ischemia/reperfusion injury [14]. Therefore, pharmacologically active reduced thiols derived from Cys are effectively delivered to the kidney proximal tubule where they downregulate these inflammatory factors via ROS scavenging. This process prevents leukocyte activation in renal ischemia/reperfusion injury. Ser-PAMAM-Cys may also directly protect renal tissue against ROS generated from the proximal tubule by scavenging it and preventing it from diffusing into the distal renal tubule. NGAL, an index of distal tubule damage, was significantly downregulated by intravenous Ser-PAMAM-Cys administration. These results suggest that Ser-PAMAM-Cys was a kidney-targeting ROS scavenger which blocked the ROS-mediated inflammatory cascade and prevented renal ischemia/reperfusion injury. Further experimentation is required to elucidate the mechanism of Ser-PAMAM-Cys-mediated renal ischemia/reperfusion injury prevention. Nevertheless, the present findings indicate that Ser-PAMAM-Cys holds promise as a kidney-targeting ROS scavenger.

## Figures and Tables

**Table 1:** Pharmacokinetic parameters of Ser-PAMAM-Cys.

Compound	Dose mg kg^−1^	AUC % of dose (h mL^−1^)	Clearance (mL h^−1^)		
			Total	Liver	Kidney
Ser-PAMAM-Cys	1.0	28.4	3.52	0.09	2.86

AUC; area under plasma concentration-time curve.

## Supplementary Materials

The following are available online at http://www.mdpi.com/1999-4923/10/4/251/s1: Figure S1: MALDI-TOF spectra of l-cysteine (Cys) and l-serine (Ser)-modified, third-generation polyamidoamine dendrimer (Cysteine content 20% (A) and 40% (B)) with a *trans*-indole-3-acrylic acid matrix; Figure S2: The purity and stability of Ser-PAMAM-Cys were evaluated by 15% polyacrylamide gel electrophoresis on sodium dodecyl sulfate (SDS-PAGE) under nonreducing conditions. Lane 1, Ser-PAMAM-Cys (Cys content: 20%); Lane 2, Ser-PAMAM-Cys (Cys content: 40%); Figure S3: (A) Time courses of plasma concentration and tissue accumulation of ^111^In-labeled Ser-PAMAM-Cys with a high degree of Cys modification (Ser content: 60%; Cys content: 40%) after intravenous administration at 1 mg kg^−1^. (B) Ex vivo imaging of NIR-labeled Ser-PAMAM-Cys with a high degree of Cys modification (Ser content: 60%; Cys content: 40%) 60 min after intravenous injection.
