# Supplementary material for: l-Cysteine and l-Serine Modified Dendrimer with Multiple Reduced Thiols as a Kidney-Targeting Reactive Oxygen Species Scavenger to Prevent Renal Ischemia/Reperfusion Injury
Source: Pharmaceutics. 2018 Dec 1;10(4):251. doi: 10.3390/pharmaceutics10040251 (PMC6321508; doi:10.3390/pharmaceutics10040251)
Supplement: Supplementary file 1 [file pharmaceutics-10-00251-s001.pdf]

pharmaceutics

MDPI

Supplementary Materials: L-Cysteine and L-Serine Modified Dendrimer with Multiple Reduced Thiols as a Kidney-Targeting Reactive Oxygen Species Scavenger to Prevent Renal Ischemia/Reperfusion Injury

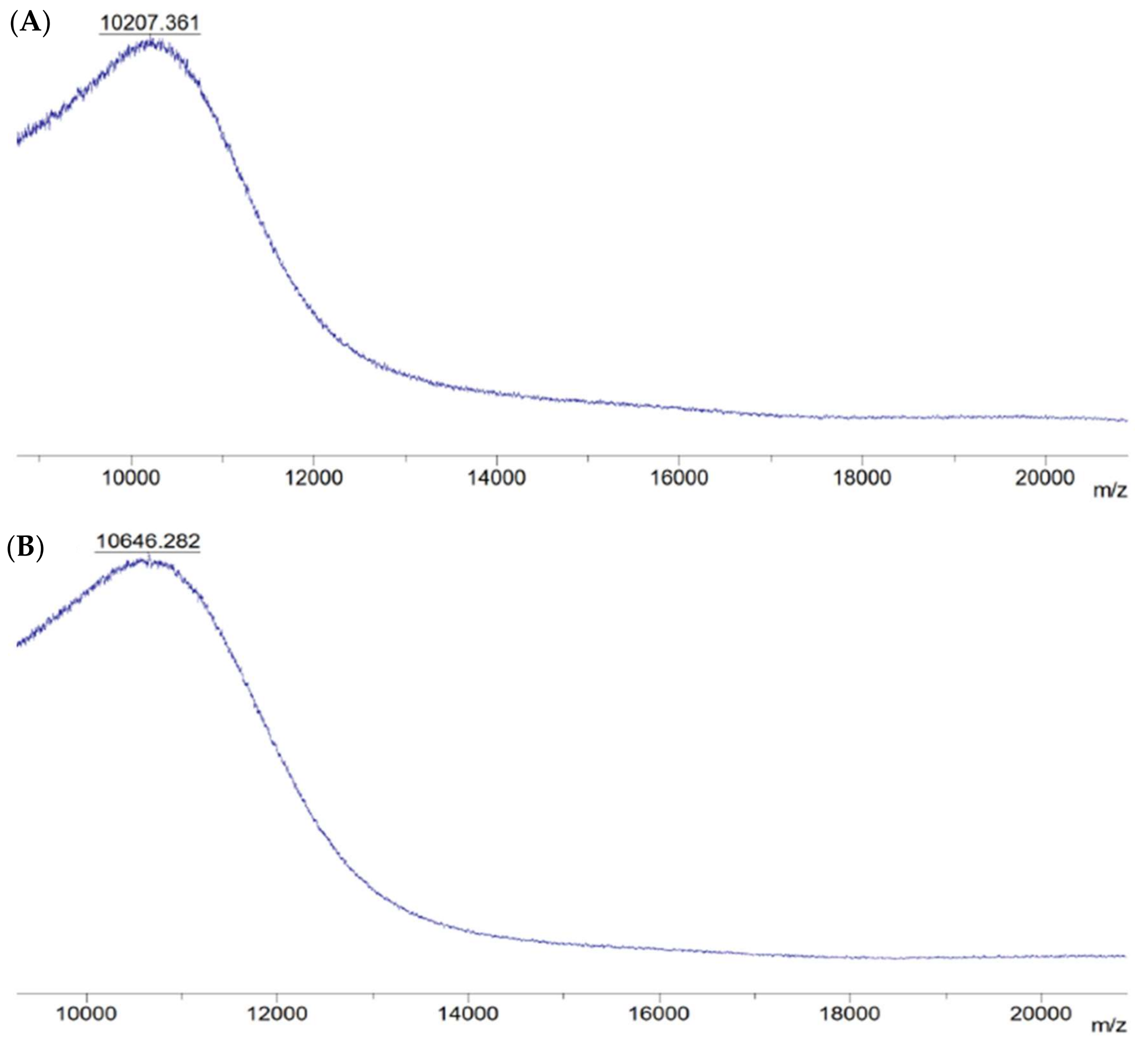

Figure S1. MALDI-TOF spectra of L-cysteine (Cys) and L-serine (Ser)-modified, third-generation polyamidoamine dendrimer [Ser-PAMAM-Cys] (Cysteine content 20% (**A**) and 40% (**B**)) with a *trans*-indole-3-acrylic acid matrix.

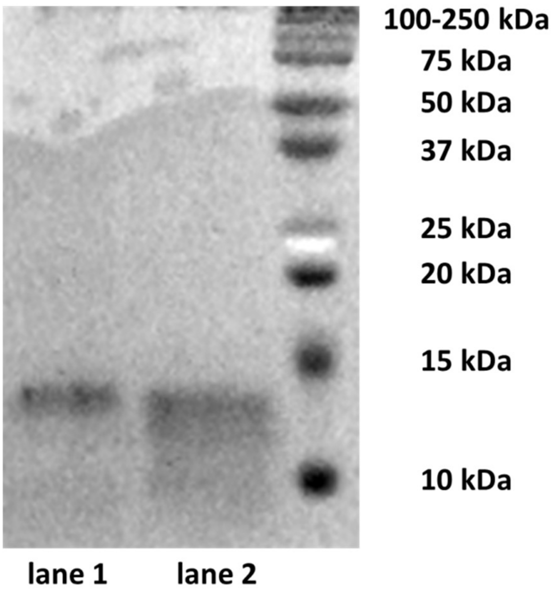

Figure S2. The purity and stability of Ser-PAMAM-Cys were evaluated by 15% polyacrylamide gel electrophoresis on sodium dodecyl sulfate (SDS-PAGE) under nonreducing conditions. Lane 1, Ser-PAMAM-Cys (Cys content: 20%); Lane 2, Ser-PAMAM-Cys (Cys content: 40%).

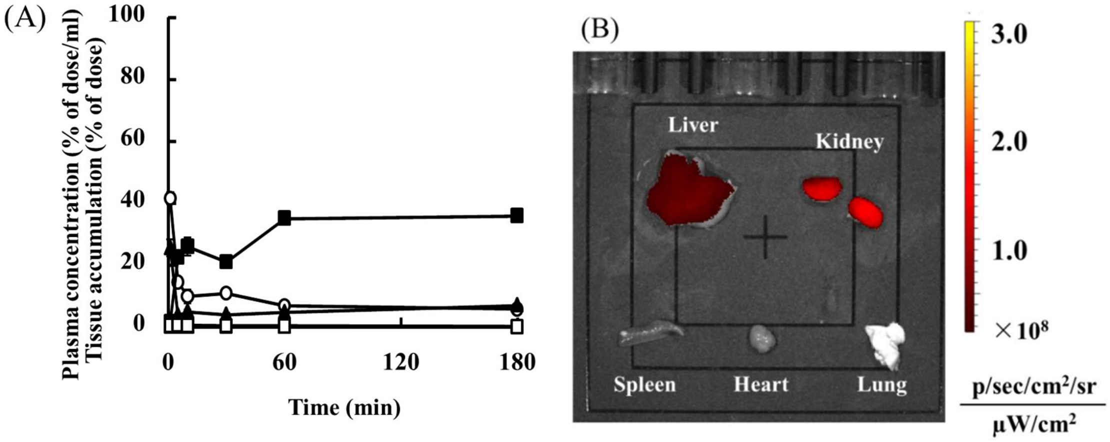

Figure S3. (**A**) Time courses of plasma concentration and tissue accumulation of ^{111}In-labeled Ser-PAMAM-Cys with a high degree of Cys modification (Ser content: 60%; Cys content: 40%) after intravenous administration at 1 mg kg^{-1}. Results are expressed as means ± SE for three mice. ○, plasma; ▲, liver; ■, kidney; ◊, spleen; Δ, heart; □, lung. (**B**) Ex vivo imaging of NIR-labeled Ser-PAMAM-Cys with a high degree of Cys modification (Ser content: 60%; Cys content: 40%) 60 min after intravenous injection. The fluorescence intensities were determined for the liver, kidney, spleen, heart, and lung.
